# Optical Waveguide Lightmode Spectroscopy (OWLS) as a Sensor for Thin Film and Quantum Dot Corrosion

**DOI:** 10.3390/s121217330

**Published:** 2012-12-13

**Authors:** Hao Yu, Carrick M. Eggleston, Jiajun Chen, Wenyong Wang, Qilin Dai, Jinke Tang

**Affiliations:** 1 Department of Chemical and Petroleum Engineering, University of Wyoming, Laramie, WY 82071, USA; E-Mail: hyu1@uwyo.edu; 2 Department of Geology and Geophysics, University of Wyoming, Laramie, WY 82071, USA; 3 Department of Physics and Astronomy, University of Wyoming, Laramie, WY 82071, USA; E-Mails: jchen12@uwyo.edu (J.C.); wwang5@uwyo.edu (W.W.); qdai1@uwyo.edu (Q.D.); jtang2@uyow.edu (J.T.)

**Keywords:** OWLS, AFM, waveguide sensors, thin films, quantum dots, ZTO, CdSe

## Abstract

Optical waveguide lightmode spectroscopy (OWLS) is usually applied as a biosensor system to the sorption-desorption of proteins to waveguide surfaces. Here, we show that OWLS can be used to monitor the quality of oxide thin film materials and of coatings of pulsed laser deposition synthesized CdSe quantum dots (QDs) intended for solar energy applications. In addition to changes in data treatment and experimental procedure, oxide- or QD-coated waveguide sensors must be synthesized. We synthesized zinc stannate (Zn_2_SnO_4_) coated (Si,Ti)O_2_ waveguide sensors, and used OWLS to monitor the relative mass of the film over time. Films lost mass over time, though at different rates due to variation in fluid flow and its physical effect on removal of film material. The Pulsed Laser Deposition (PLD) technique was used to deposit CdSe QD coatings on waveguides. Sensors exposed to pH 2 solution lost mass over time in an expected, roughly exponential manner. Sensors at pH 10, in contrast, were stable over time. Results were confirmed with atomic force microscopy imaging. Limiting factors in the use of OWLS in this manner include limitations on the annealing temperature that maybe used to synthesize the oxide film, and limitations on the thickness of the film to be studied. Nevertheless, the technique overcomes a number of difficulties in monitoring the quality of thin films *in-situ* in liquid environments.

## 1. Introduction

With the development of thin film and nanoparticle synthesis techniques, the ability to characterize and monitor such materials over time has increased in importance. In some applications, thin nanocrystalline films may be subjected to corrosive liquid environments. In dye-sensitized solar cells or quantum dot sensitized solar cells [1–3], the substrate material (often an oxide semiconductor) or the quantum dots (QDs) can be subjected to harsh liquid environments. There are important questions about the long-term stability of substrate and QD materials in solutions that may reach 80 °C or more in field deployment [4]. This is especially true in the case of multicomponent oxides because one component may react or leach out of the material faster than the other, creating an altered residual solid lacking the optimal photoelectrochemical characteristics of the original material [5–7]. Over time, QDs and their attachment to the substrate may also be compromised. Sensor systems that allow monitoring of thin film and nanoparticulate materials in liquid environments are needed for the experimental assessment of long-term chemical stability of such solar cell materials.

It is often simple to use *ex-situ* techniques such as electron microscopy or electron spectroscopy in the study of solid alteration in liquid environments. The need to dehydrate the material to be studied, however, imposes the risk that the structures to be observed will be altered by dehydration. Drying a sample for (or upon) insertion into a vacuum can involve the evaporative shrinkage of liquid droplets whose surface tension “sweeps up” and concentrates loosely attached surface particles. Drying can leave a residue of crystallized salts [8]. This can be an important consideration in the case of surfaces decorated with QDs. Chemical leaching of solid materials can create a hydrated layer whose structure can be drastically altered by dehydration in air or in vacuum [9]. Therefore, *in situ* techniques that allow routine monitoring of thin-film and nanocrystalline mass on surfaces are needed.

Atomic force microscopy (AFM) is capable of high resolution in a liquid environment, but translating AFM data into quantitative assessment of thin film characteristics can be difficult. For example, AFM cannot easily be used to quantify the mass density (in units of, e.g., nanograms per square centimeter, ng·cm^−2^) or changes in mass density of an oxide film or of QDs over time. There are several biosensor techniques that are often used to monitor surfaces for the thickness or mass of biofilms or sorbed biomolecules (generally proteins) in-situ, including ellipsometry (ELM), the quartz crystal microbalance (QCM), and optical waveguide lightmode spectroscopy (OWLS) [10–12].

In this study, we show how OWLS can be modified to allow monitoring of inorganic material films and quantum dots *in-situ*. As mentioned above, our application is the study of the chemical stability of quantum dot sensitized solar cells based on zinc stannate (zinc-tin-oxide, ZTO, Zn_2_SnO_4_) substrates [13]. Because ZTO has a high refractive index (the refractive index of ZTO has been reported as 2.03 at 633 nm [14]), it is well suited to OWLS in the form of a thin layer on a waveguide surface (see next section). Likewise, the quantum dots have size and refractive index characteristics similar to that of proteins more commonly studied using OWLS. Although the solar devices made with ZTO substrates use nanowires, we emphasize that the ZTO thin films were made for the specific purpose of corrosion studies using OWLS.

## 2. Methods

### 2.1. Optical Waveguide Lightmode Spectroscopy (OWLS)

**Figure 1.**
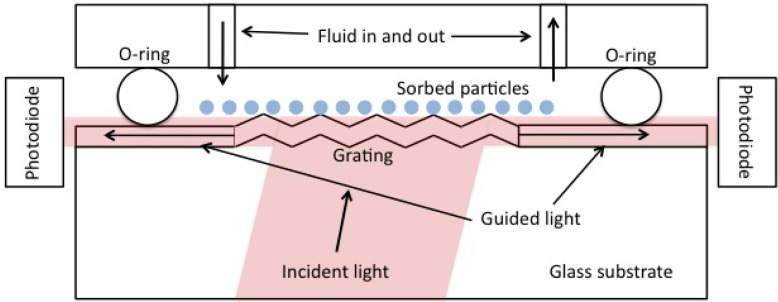
The physical configuration of OWLS. The angle at which incident light is guided with constructive interference to the photodiode detectors is used to calculate the effective refractive index of the waveguide (see text).

We used an OWLS 120 instrument (Microvacuum, Budapest, Hungary) to measure the mass density of ZTO coatings and pulsed laser deposition (PLD) derived CdSe quantum dots on waveguide surfaces. OWLS is described in detail elsewhere [11,15]. In brief, plane-polarized 633 nm laser light is coupled into a waveguide of approximately 200 nm thickness (range reported by the manufacturer, is 170 to 220 nm) by means of a grating embossed on the waveguide. Under conditions of total internal reflection within the waveguide, light can only traverse the waveguide and be detected if constructive interference occurs. Angles of constructive interference are sensitive to the effective refractive index (*N*) of the waveguide according to *N = nsinα* + *lλ*/*Λ* where *n* is the refractive index of air (or other covering medium), λ is the laser wavelength (633 nm), Λ is the grating constant (417 nm for our waveguides), and *l* is the diffraction order. *N* depends on the mass of sorbed molecules or material in a coating layer. Figure 1 shows the basic physical configuration of OWLS.

Typically, OWLS is used to quantify the adsorption of proteins to waveguide surfaces [16]. There is an implicit assumption that the characteristics of the waveguide do not change over time. One of the key advantages of OWLS is that very small quantities of QDs or other materials are needed in order to obtain meaningful datasets.

Because the OWLS technique relies upon total internal reflection (TIR) in a waveguide material of relatively high refractive index (1.77 ± 0.03), materials that have higher refractive index can be used to coat the waveguide in a thin layer and thus allow for polarized light to exit the original waveguide, undergo TIR at the coating-liquid interface, and re-enter the waveguide. The light that traverses the waveguide thus does so at a precise angle that reflects both the properties of the coating layer as well as sorption/desorption at the layer-liquid interface. The high refractive index of ZTO makes it a good material for coating OWLS waveguides. The original OWLS waveguides used in this study are of composition Si_x_Ti_(1−x)_O_2_ where x = 0.25 ± 0.05 [17].

In OWLS, it is usually necessary to achieve a stable baseline in a particle-free buffer solution before introducing buffer solution containing particles (or, more typically, proteins). Sorption is monitored until a satisfactory data set is recorded, and then particle-free solvent is reintroduced and desorption is monitored. Because OWLS depends on refractive index contrast between particle-free and particle-containing solvent, the measured adsorbed mass includes the mass of particles but not that of the solvent between the sorbed particles. Determination of sorbed mass in OWLS requires knowledge of dn/dc, the change in refractive index of a solution with a change in mass concentration of the sorbing solute. We measured this quantity, and also calculated it for our different solutions based on the refractive index of suspended particles mixed with the refractive index of water in proportion to concentration. We found that this quantity was nearly identical to that typically used for proteins and that it therefore had negligible effects on our results compared to other uncertainties.

When monitoring sorption of particles or proteins, we follow the manufacturer’s recommended baseline stability criterion of less than 10^−8^ s^−1^ drift in the transverse magnetic effective refractive index value prior to initiating the adsorption phase of each experiment. In data processing, the baseline is chosen manually to represent zero sorbed mass. This method, for example, can be used in OWLS monitoring of quantum dot attachment to surfaces. However, in most of the experiments reported here we were forced to use different baseline procedures. In order to monitor mass loss from thin ZTO films, we cannot use a pre-experiment baseline because the ZTO film deposition process involves annealing the ZTO (see the next section) along with the waveguide it is deposited upon. We cannot assume that the original waveguide, after ZTO deposition and annealing, has the same properties as the waveguide after deposition.

It is important to remember that the choice of baseline in OWLS is arbitrary, based usually upon time-invariant data for which it is known that no particles or proteins have yet sorbed to the surface. OWLS observations are then interpreted in terms of mass gain upon introduction of particles or proteins to the solution and their subsequent sorption to the surface. The choice of baseline does not affect the absolute mass change measurement, merely the zero point. Baseline only has significance as a point of known mass coverage against which to compare changes. In this study, the only way to obtain a baseline for the annealed waveguide *after* ZTO film deposition, assuming that the annealing step (see Section 2.2) changes the original waveguide properties, is to use post-experiment time-invariant data. In addition, we can independently confirm mass-loss using AFM observations of the film.

### 2.2. Preparation of ZTO-Coated Waveguides

The ZTO films for OWLS analysis were prepared by a sol-gel method. The precursor solution for 100-nm-thick films contained 0.16 M tin 2-ethyhexanoate (Alfa Aesar, 96%) and 0.33 M zinc acetate (Alfa Aesar, 98%) dissolved in absolute ethanol using 0.5 M triethanolamine (Alfa Aesar, 98%) as chelating agent. The solution can be diluted further for thinner films and the film thickness is proportional to the concentration. The solution was stirred magnetically for 2 h at 50 °C. The transparent precursor solution was then aged for 48 h. Spin-coating at 4,000 rpm for 120 s was used to apply the precursor onto an OWLS waveguide. After spin-coating, the waveguide was annealed at 500 °C for 4 h. This temperature was chosen because it is the upper temperature limit for the survival of the waveguide through the annealing process without loss of waveguide quality. As the results show, the annealing step was insufficient to form a film that did not detach from the waveguide in aqueous solution. Nevertheless, the films were very useful for demonstrating the modified OWLS sensor system for monitoring film integrity.

**Figure 2.**
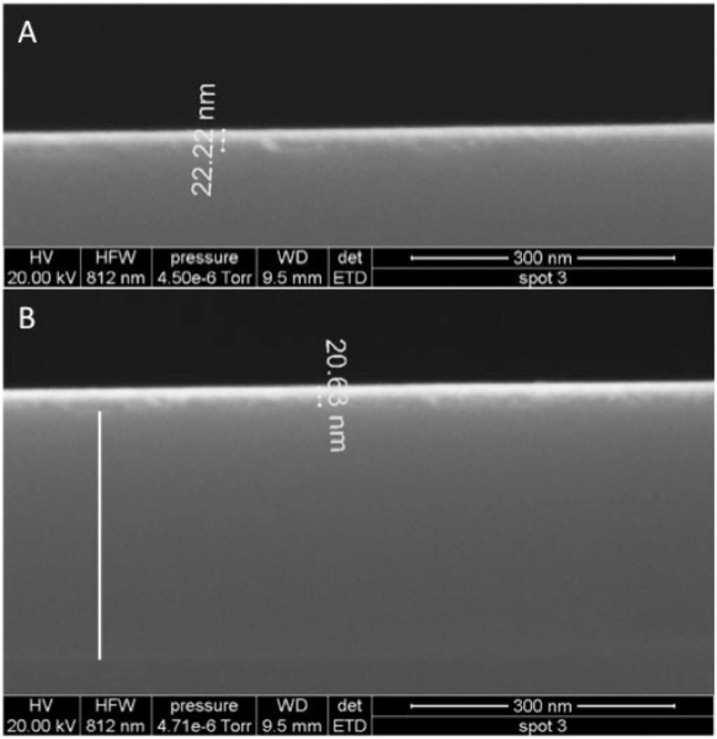
Field emission SEM images of ZTO waveguide coatings. (**A**) A 22–23 nm ZTO coating (composition confirmed using energy dispersive X-ray analysis, data not shown). (**B**) A 21 nm coating made on a waveguide; the white line shows the thickness of the waveguide material.

Films were imaged with field emission scanning electron microscopy (FESEM) using an FEI instrument (images and imaging conditions in Figure 2).

**Figure 3.**
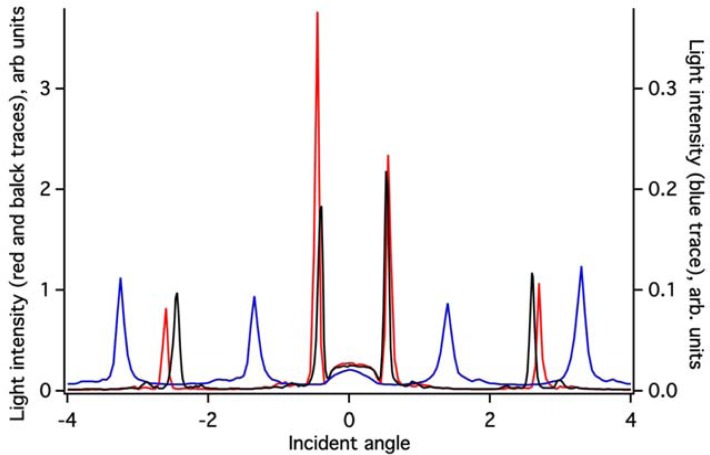
OWLS spectra for a clean (Si,Ti)O_2_ waveguide (red trace) and for the waveguide after deposition of 21 nm of ZTO (blue trace). Note the attenuation of the light signal, and the large change in position of the peaks due to the mass of ZTO deposited. After the removal of the film during an experiment (see text below), the spectrum from the uncovered waveguide (black trace) is similar to, but not the same as, the spectrum from the original un-annealed waveguide.

The ZTO coating on the (Si,Ti)O_2_ waveguide surface greatly attenuates the intensity of light detected in the OWLS spectrum (Figure 3). Because the ZTO has a higher refractive index than the underlying waveguide, light that reaches the ZTO-waveguide interface refracts into the ZTO. Light can then undergo TIR at the ZTO-solution interface and re-enter the waveguide. However, much of the light that enters the ZTO is evidently scattered out of the ZTO and never re-enters the waveguide, resulting in signal attenuation. However, as Figure 3 shows, the ZTO-coated waveguide OWLS spectrum still exhibits clear peaks at precise angles. These are used to determine sorbed mass. As long as the peak position can be determined, peak height does not matter. Note that in Figure 3, the peaks in the spectrum for the ZTO-coated waveguide occur at very different angles compared to the original uncoated waveguide. This is a direct consequence of deposition of the ZTO layer and its effect upon the effective refractive index of the waveguide sensor.

### 2.3. Preparation of Quantum Dot Coatings by Pulsed Laser Deposition

CdSe quantum dots (QDs) were deposited on a waveguide using a pulsed laser deposition method. A Nd:YAG laser with a frequency-quadrupled wavelength of 266 nm, pulse repetition rate of 10 Hz and 6.4 J/cm^2^ laser fluence, was used for the deposition. Fluence was calculated by dividing the laser output energies by the ablation spot sizes on the solid CdSe target. The deposition was carried out in a vacuum chamber at a pressure of 10^−6^ torr. A fused quartz window allowed the UV laser beam to pass through. The laser beam was focused using a lens with a focal length of 30 cm located in a position outside the chamber such that it focuses the laser energy on the target positioned inside the chamber. The target was a CdSe bulk piece purchased from Alfa Aesar. Material from the target is then deposited on the waveguide substrate placed in the vacuum chamber 6 cm from the target. The laser was focused on the target for 15 minutes.

### 2.4. Aqueous Solutions

The solutions used in experiments with ZTO films were 10 mM 3-(N-morpholino)propanesulfonic acid (MOPS) at a of pH of 7.0. The borate buffer for pH 10 solutions is made from 10 mM Na_2_B_4_O_7_•10H_2_O adjusted to pH 10 with 0.1 M NaOH. We also used a 0.006% solution of 3-mercaptoproprionic acid (MPA) as a surface preparation that promotes sorption of CdSe quantum dots to waveguide surfaces. Because the sorption of materials such as quantum dots is a “normal” use of OWLS, we do not report those results here. However, our QD-containing solutions had pH values of 6.2 in one case and 10.3 in another case (caused by residual tetramethylammonium hydroxide from QD preparation).

## 3. Results

### 3.1. ZTO Coated OWLS Sensors

**Figure 4.**
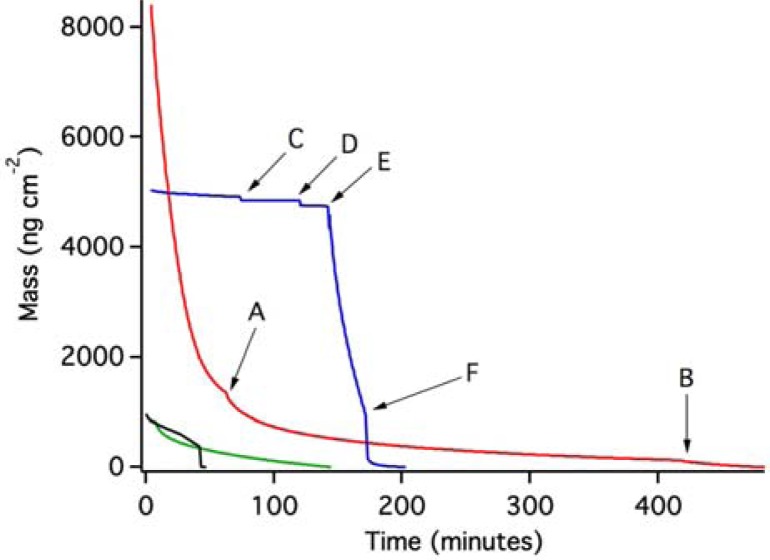
OWLS results from four trials of the ZTO-coated waveguides. All data is presented with a post-experiment baseline. The red trace represents an experiment in which a ZTO-coated waveguide was exposed to pH 7 MOPS buffer, and points A and B show when fresh buffer was injected into the OWLS fluid cell. For the blue trace, the experiment started and ended with injections of pH 7 MOPS buffer, with other injections (points C through F) described in the text. The green trace (pH 7 MOPS buffer) and black trace (pH 10.3 solution, 2nd injection of solution where the data drops suddenly) show incomplete removal of a ZTO layer. Apparent complete loss of the ZTO coating (red and blue traces) is confirmed in AFM imaging.

Figure 4 shows the results of OWLS experiments in which we have used post-experiment data as a baseline against which to compare mass loss from the waveguide due to the breakdown and removal of the ZTO film in aqueous solutions.

The red trace in Figure 4 shows the result of an experiment in which pH 7 MOPS buffer was introduced to the OWLS fluid cell. Mass loss commenced immediately and was slightly accelerated with each of two further injections of the MOPS buffer at points A and B. Overall, mass coverage was reduced by 8,413 ng·cm^−2^. A second experiment (blue trace, Figure 4) also started with pH 7 MOPS buffer, and a stable baseline was initially achieved. At point C, a 0.006% MPA-containing MOPS solution resulted in a sudden small drop in mass. Reinjection of the same solution at point D resulted in another small mass loss. QD-containing solution (pH 6.31) was injected at point E, which resulted in very rapid and extensive apparent mass loss at a rate similar to that in the initial part of the red trace. Correcting for pH change would increase the apparent mass loss slightly [18]; however, this correction was not necessary. QD-free MOPS buffer (pH 7) was reinjected at point F, creating conditions identical to the starting conditions. This resulted in renewed mass loss, followed by stabilization at a total mass loss of about 4,910 ng·cm^−2^ compared to the starting point under the same aqueous conditions.

Two other experiments were conducted, one in MOPS buffer (green trace) and one at pH 10.3 (black trace; see methods section). In both cases, apparent mass loss occurred (the experiments were terminated prior to complete loss of the ZTO layer).

**Figure 5.**
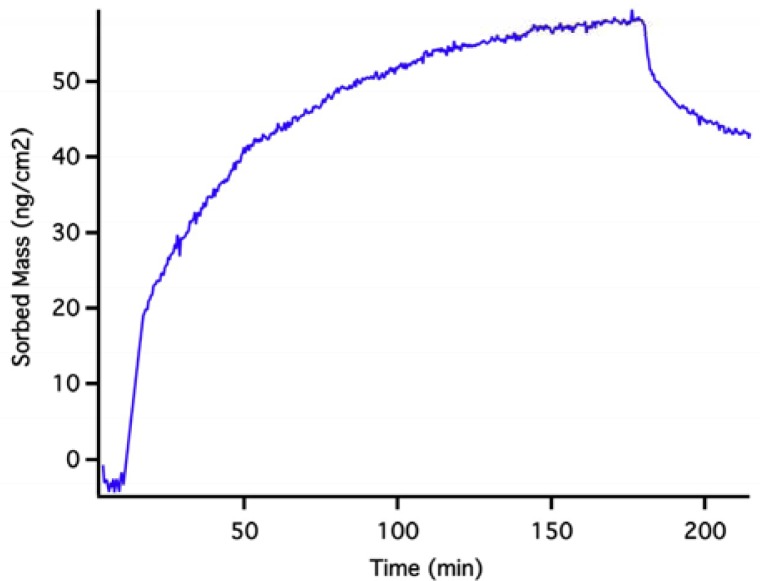
OWLS data for the sorption of MPA to a waveguide surface from a pH 7 solution buffered with MOPS (see text). At ∼180 min, the mass drops due to desorption of MPA when MPA-free buffer is injected.

Figure 5 shows the effect of injection of the MPA-MOPS solution for a waveguide previously equilibrated with pH 7 MOPS buffer using a waveguide with no ZTO coating. In this case, MPA sorbs to the oxide waveguide surface via inner-sphere bonding between the carboxylic functional group on the MPA molecule and metal centers on the oxide surface. Note that in Figure 4, instead of the mass gain shown in Figure 5, mass loss is observed upon introduction of the MPA-containing MOPS solution. This is another indication that the results in Figure 4 are not caused by MPA adsorption, but instead are caused by ZTO layer detachment from the substrate.

These results require some discussion. First, the dissolution of ZTO is so slow that mass loss could not possibly have been caused by dissolution. This, and the differences between experiments in Figure 4, led us to conclude that the ZTO coating was being removed from the surface without dissolving. The ZTO coatings were all made using the same procedure, but this does not preclude variations in the thickness, cohesion, and properties of the coating. The physical treatment of the coated waveguide is probably important as well. Manual injection of fluids into the OWLS fluid cell can result in different fluid flow velocities during different injections, leading to differences in the subsequent cohesion of the coating. In addition, the surface tension forces of air-water interfaces could readily disrupt a poorly cohesive coating if bubbles are pushed through the fluid cell.

Second, we point out that a 20 nm thick uniform (nonporous) ZTO film would have a mass coverage of 12,860 ng·cm^−2^. In Figure 4, mass loss of over 8,400 ng·cm^−2^ is observed in one case, and almost 5,000 ng·cm^−2^ in another case. Both experiments ended with a time-invariant flat-line potentially representing complete loss of the ZTO coating from the surface, exposing the underlying waveguide. This interpretation was confirmed using AFM (see below). The end of each experiment thus constitutes a defensible baseline against which to measure mass loss, even if variations in pH and other conditions occurred during the course of an experiment (we assume that the other two experiments shown in Figure 4, with smaller amounts of mass loss, show removal of only a small portion of the ZTO layer).

**Figure 6.**
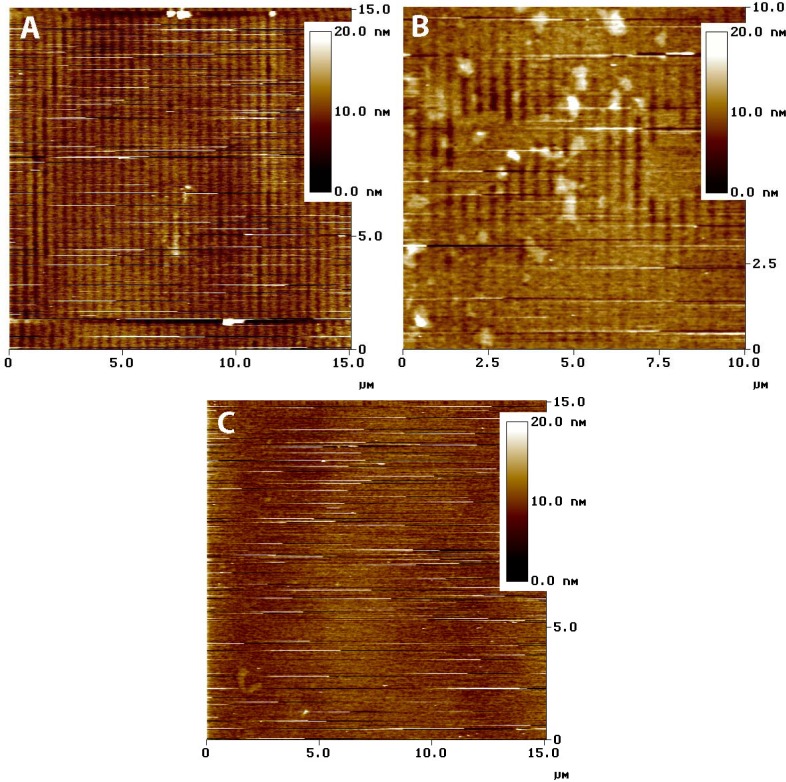
Tapping mode AFM images of the originally ZTO-coated waveguide recovered from the experiment are shown in Figure 4, timepoints C through F. (**A**) image taken in the middle of the area of the waveguide covered by the fluid cell. (**B**) Image taken from the edge of the fluid-covered area. (**C**) Image taken from outside the fluid covered area. Vertical color scales are given as insets, and the image size is given in μm.

If the entire ZTO layer was removed, the ratio of mass lost to the mass of a hypothetical uniform nonporous film of the same thickness provides an estimate of film porosity. The results in Figure 4, indicate porosities of about 35% and 62% for the red and blue traces, respectively. AFM imaging was used to assess the state of the ZTO-coated waveguides with these layer thicknesses in mind. Figure 6 presents a summary of AFM results, which indicate a layer with significant surface roughness and probably with significant porosity. Although the calculated porosities would be high for bulk porous solid, they are not remarkable given the surface roughness of the layer in Figure 6.

Contact-mode AFM imaging of fresh ZTO-coated waveguide surfaces resulted in physical removal of the ZTO material (data not shown), a result consistent with weak adhesion of the film to the waveguide substrate. Tapping mode AFM imaging was relatively stable (Figure 6) but the frequent streaks in the images are the result of dislodged particles interfering with tip motion. Figure 6(A) shows the waveguide surface near the middle of the fluid covered area. The waveguide grating is readily visible, indicating that the covering ZTO layer has mostly been lost. Figure 6(C) shows the waveguide near the edge of the fluid-covered area; patches of ZTO-covered area occur along with uncovered areas showing the waveguide diffraction grating. Larger aggregate particles are also visible, which we speculate result from aggregation of smaller fundamental ZTO particles during mobilization of the particle layer by fluid flow. Only in the area not exposed to solution (Figure 6(C)) is the ZTO layer intact, though with frequent streaking indicating that ZTO particles are easily dislodged from the layer. The AFM results confirm the OWLS observation that ZTO was removed from the waveguide surface upon injection of the QD-containing solution.

### 3.2. PLD QD Coated OWLS Sensors

**Figure 7.**
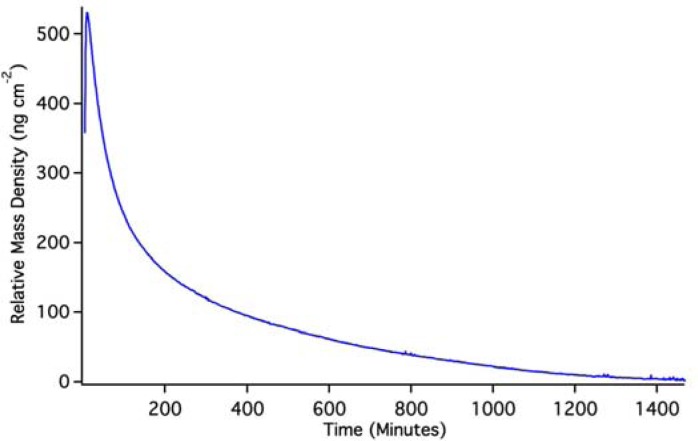
OWLS data for the dissolution of PLD QDs during a ∼1-day period in pH 2 HCl. As discussed in the text, this reflects an extrapolated starting point of about 630 ng·cm^−2^.

OWLS results for (Si,Ti)O_2_ waveguides coated with PLD QDs are shown in Figure 7. Again, because the QDs were pre-attached to the waveguide surface, it is impossible to achieve a pre-QD baseline in solution. Changes in mass density indicated by OWLS are therefore based on a post-run baseline. To achieve such a baseline, we used a pH 2 HCl solution to dissolve QDs off of the waveguide surface.

The initial rise in mass is the result of the solution initiating an approach to a baseline that can be predicted by exponential extrapolation to occur at about 640 ng·cm^−2^. The OWLS signal then reaches a peak and descends due to dissolution of PLS QDs in the pH 2 solution. Back-extrapolation of this mass loss using a double-exponential fit indicates an original starting mass density of 620 ng·cm^−2^, in good agreement with extrapolation of the initial rise. This suggests that, over the course of the experiment, we have dissolved the equivalent of 630 ± 10 ng·cm^−2^ quantum dots from the waveguide surface. A full monolayer of hexagonally close packed 5 nm quantum dots would have a mass coverage of about 3,500 ng·cm^−2^, so the PLD QD density must be about 18% of a full monolayer assuming complete dissolution. We are confident that the mass loss signal in this case stems from dissolution because it is known that un-capped quantum dots dissolve on this time scale [19] (which extends for 1 day).

**Figure 8.**
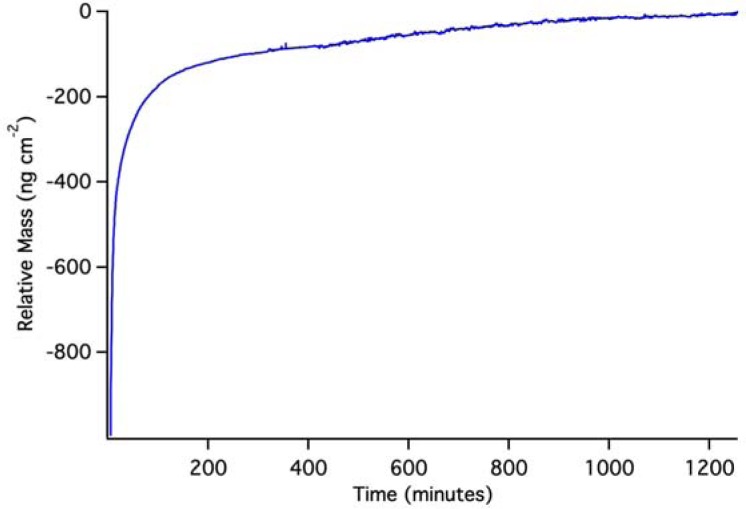
OWLS results for a PLD QD layer on a waveguide at pH 10 (borate buffer). No dissolution is observed; the apparent mass change is simply due to the approach to baseline typical of initial contact of an OWLS waveguide sensor with buffer solution.

Figure 8 shows the behavior of a PLD QD layer in pH 10.0 borate buffer. In this case, there is no obvious dissolution. Instead, the OWLS signal reaches a stable long term baseline over a day-long period. This result does not mean that the PLD QD layer is ∼900 ng·cm^−2^; it simply shows that the QD layer is stable and does not lose mass from the surface. Remembering that the PLD QD layer starts out dry, the sorption and filling of pore space in the waveguide by solvent causes the apparent mass rise.

## 4. Conclusions

Here, we show that OWLS can be used to monitor the stability of thin film materials and of quantum dot layers over time. This application of OWLS differs from application to protein sorption in several ways. The technique involves the construction and modification of waveguide sensors with thin films or quantum dots. The OWLS data must often be treated differently than in normal OWLS applications because a stable baseline often cannot be achieved prior to the experiment. Remembering that a stable baseline has no effect upon the relative mass change measurements, simply on the zero point, we used a post-experiment baseline for which we know the end result (for example, loss of ZTO or QD film). Exponential or double exponential extrapolation of OWLS data can provide reasonable estimates of the original mass coverage prior to the initiation of dissolution. In some cases, when one cannot be assured of the long-term stability of a coating on a substrate or of changes in the substrate itself, a stable baseline cannot be achieved. In such cases, while quantitative mass coverage changes cannot be clearly quantified, relative changes in mass still contain qualitative information about the integrity of thin films. In addition, AFM and OWLS are complementary techniques that can be used in-situ in a variety of fluids to test for consistency between mass gain and mass loss and the topographic surface landscape that results from such gain or loss. For non solid-state solar cells in particular, the stability and longevity of thin films and quantum dots on surfaces in a liquid environment is key to the viability of any new solar technology. Modified OWLS sensors represent one relatively simple bench-top method for assessment of film integrity.
